# Direct Examination of the Larynx, Trachea, and Œsophagus

**Published:** 1909-01-23

**Authors:** 


### Laryngology and Rhinology.

### DIRECT EXAMINATION OF THE LARYNX, TRACHEA, AND ŒSOPHAGUS.

Recently great advances have been made in the development of methods designed to give access for inspection and even manipulation to deeply situated regions of the interior of the body. The urethroscope, the cystoscope, and the sigmoidoscope have all been so greatly improved in point of technical detail that they have largely replaced the older and more uncertain methods of diagnosis in obscure cases, and have become essential for the treatment of many forms of disease in their respective regions. When the advantages of any method are once firmly established it is the pride of surgery that merely technical difficulties seldom remain for long unsurmounted. Modified to suit the various regions the fundamental principle of these methods of examination is the same, namely, to apply a brilliant illumination to the end of a tube which can be introduced into the organ to be examined; and it has been found that a straight tube can in practice be introduced into the various canals of the body without much difficulty, so flexible and elastic are the tissues. The application of this principle to examination of the larynx, trachea, bronchi, and œsophagus has been somewhat delayed, partly because we have already in the laryngoscopic mirror a method which is very efficient for most cases of laryngeal disease, and partly because the technical difficulties of the direct method are specially great in this region, on account of the existence of powerful reflexes designed expressly to prevent the introduction of foreign bodies. Nevertheless tentative experiments in direct laryngoscopy were made many years ago, but it is only quite recently that the difficulties of technique have been so conquered as to make the method of any real practical value. The story of its evolution is interesting.

It has long been known that the epiglottis comes into view on firmly depressing the tongue, and Kirstein found that by depressing further it was possible to obtain a view of the arytenoids and posterior part of the glottis. For this method, which he called "autoscopy," he employed a strong tongue-depressor of extra length slightly bent downwards at the tip; the patient sat with the body bent forwards and the head extended and the instrument was introduced into the glosso-epiglottic fossa when firm pressure downwards and forwards on the tongue gave the view required. The direct view is of great advantage for such delicate manipulations as removal of a papilloma from the vocal cords, but unfortunately the anterior parts of the larynx and the greater part of the cords do not come into view, and this method was never really satisfactory. To enable the instrument to be brought into a more vertical position, and so to give a view of the front of the larynx, Kirstein added a kind of metal arch over the instrument, converting the proximal end into a tube, and thereby insuring a space for examination between the spatula and the upper teeth, but still the method was not generally adopted. The illumination was unsatisfactory; the ordinary forehead mirror was employed or else Kirstein's electric forehead lamp in which the light is reflected by a small perforated mirror placed at an angle. But the illuminant in these methods is too far from the end of the tube, it is not brilliant enough, and it is displaced by every movement of the tube or the head. After a considerable interval Killian introduced a series of tubular specula of different sizes and lengths mounted at a right angle on a strong handle, and designed for introduction into the larynx, treachea, and œsophagus. Stimulated by the advances which had in the meanwhile been made in the methods of examination in other parts of the body, laryngologists all over the world began to work with these instruments and to improve the technique.

It was found that if the patient sits in the upright position with the head thrown very far back, it is possible to pass a tubular speculum directly into the larynx without much difficulty and without great discomfort after a very thorough application of cocaine; if under general anæsthesia the patient is placed on the back with the head over the end of the table, or on the side with the head fully extended. Tubes were passed down the trachea and into the bronchi either by the mouth or through a tracheotomy opening, and the medical journals were full of accounts of removal of foreign bodies by this method until they became too common to record. There were still many technical imperfections, notably in the illumination, the narrowness of the laryngeal tubes, and the difficulties of dealing with saliva, mucus, and blood. These have been gradually eliminated and in the newest patterns, such as that designed by Brünings, we have a very valuable instrument. In this form the lamp is placed on the instrument, at a right angle to the tube, and the light is reflected by a small plane mirror with a perforation in the centre for the eye of the observer. The focussing lens is movable and the mirror can be accurately centred by means of two screws. The lamp and mirror can be moved away in a line with the tube, which is still illuminated, so that there is room for the passage of instruments. The larnygeal tubes are cut away so as to be mere spatulæ at the distal end, and, in fact, approach the shape of the Kirstein instrument, thus giving a wide view. The tracheal tubes are fitted with telescopic extensions for introduction into the bronchi and can be pushed out under full illumination and inspection, and a saliva pump is provided for the removal of fluids.

No doubt the direct method will be largely used in the future. It is essential for the removal of foreign bodies in the air tubes, replacing the haphazard methods of groping with forceps and probang, and the very dangerous attempts at removal through the chest wall which were formerly the only hope in cases of bodies impacted in the bronchi. It has greatly enlarged our methods of diagnosis and treatment of disease of the trachea and œsophagus. It will not replace the laryngoscope for examination of the larynx nor for the simpler intralaryngeal manipulations, for it is not so comfortable for the patient; but for the more difficult cases it will prove invaluable. Previously the only method of treating papillomata of the larynx in children was by a lengthy course of training or, when this was impossible, by combined cocaine and chloroform anæsthesia in the erect position. They can now be removed with comparative ease without the dangers of the erect position or of the entrance of blood into the trachea. It is not a very difficult matter for one skilled in the manipulation to remove a growth from the vocal cord of an adult by the ordinary method with the laryngoscope, but this procedure is always an anxious one by reason of the possibility of damage to the cord, and sometimes it is very difficult for various reasons, such as the shape of the neck or dependence of the epiglottis; in such cases the direct method is of great value, and in general the surprisingly clear view of the parts, which it affords, renders it a most useful addition to the technique of laryngology.

